# Schistosomiasis as a disease of stem cells

**DOI:** 10.1016/j.gde.2016.06.010

**Published:** 2016-10

**Authors:** George R Wendt, James J Collins

**Affiliations:** Department of Pharmacology, UT Southwestern Medical Center, Dallas, TX 75390, United States

## Abstract

Schistosomiasis is a devastating parasitic disease caused by flatworms of the genus *Schistosoma*. The complex life cycles and developmental plasticity of these parasites have captured the attention of parsitologists for decades, yet little is known on the molecular level about the developmental underpinnings that have allowed these worms to thrive as obligate parasites. Here, we describe basic schistosome biology and highlight how understanding the functions of stem cells in these worms will transform our understanding of these parasites. Indeed, we propose that schistosomiasis is fundamentally as disease of stem cells. We hope this review will attract new interest in the basic developmental biology of these important organisms.

**Current Opinion in Genetics & Development** 2016, **40**:95–102

This review comes from a themed issue on **Cell reprogramming, regeneration and repair**

Edited by **Peter W. Reddien** and **Elly M. Tanaka**

For a complete overview see the Issue and the Editorial

Available online 5th July 2016

**http://dx.doi.org/10.1016/j.gde.2016.06.010**

0959-437X/© 2016 The Authors. Published by Elsevier Ltd. This is an open access article under the CC BY license (http://creativecommons.org/licenses/by/4.0/).

## Introduction

Schistosomes infect more than 200 million of the world's poorest people [1]. These parasites claim the lives of 250,000 people annually [2], but the chronic disability associated with infection robs millions more of the ability to live healthy and productive lives, effectively condemning infected individuals to a life of poverty [3]. To put the scope of this problem into perspective, some estimates suggest that the global morbidity due to schistosome infection may reach levels rivaling diseases including malaria, TB, and perhaps even HIV/AIDS [4]. Further, treatment of schistosomiasis relies upon a single drug (praziquantel) and it remains unclear how effective this drug will be in eradicating this disease in the developing world [5].

While the effects of the schistosome infection are horrific and new therapeutics are urgently needed, the rich, fascinating, and virtually unexplored biology of these parasites should not be ignored. In fact, recent years have seen important advances in schistosome biology, setting the stage for major progress in understanding both the organism and the disease. These advances include the publication of the genomes of the schistosome species that are major human pathogens [6, 7, 8], the development of genetic tools to map mutations in the genome [9], methods for RNA interference (RNAi) [10, 11, 12], tools for robust whole-mount *in situ* hybridization [13, 14••], a growing set of tissue specific markers [14••, 15], and promising developments in the generation of transgenic parasites [16, 17]. There is even a National Institutes of Health-supported Schistosomiasis Resource Center that provides schistosome material and training to investigators free of charge [18]. Given these resources, basic studies of these unique parasites are poised for a renaissance. Here we detail one emerging area of investigation in these parasites: the biology of stem cells. Although few molecular details about schistosome stem cells exist, there is a great deal of evidence to suggest that these cells are critical for the success of this organism as a parasite. As such, we believe that schistosomes present a fantastic model organism to ask basic questions about stem cell behavior and regulation while simultaneously addressing fundamental aspects of an important disease.

## A primer on schistosome biology

Schistosomes are members of the phylum Platyhelminthes (flatworms) which includes a myriad of free-living and parasitic taxa that inhabit most aquatic and some humid terrestrial environments [19]. Perhaps the most well-known flatworms are the free-living freshwater planarians. Capable of regenerating following nearly every type of injury, planarians employ a population of pluripotent stem cells known as neoblasts that fuel not only regeneration but also worm growth and tissue homeostasis [20, 21, 22]. Studies of planarians date back over one hundred years and with recent advances in molecular tools, these worms have enjoyed a resurgence in their use as model organisms for the study of regeneration and stem cell biology [20]. Though planarians represent a fascinating model for regenerative and developmental biology, the planarian's parasitic relatives, the Neodermata, should not be ignored. The Neodermata represent a monophyletic clade that includes all three groups of parasitic flatworms: the monogeneans, the cestodes and the trematodes [23•, 24]. The ability the Neodermata to parasitize nearly every vertebrate on earth is due in large part to their extreme developmental strategies. Monogeneans can develop like ‘Russian Dolls,’ with multiple generations of worms developing inside a single mother [25]. Cestodes (tapeworms) can grow tens of meters inside their host by perpetually adding new segments to their body, with each segment possessing sexually mature reproductive organs [19]. However, there are few developmental feats that can eclipse the remarkable life cycles exhibited by the trematodes.

**Figure 1:**
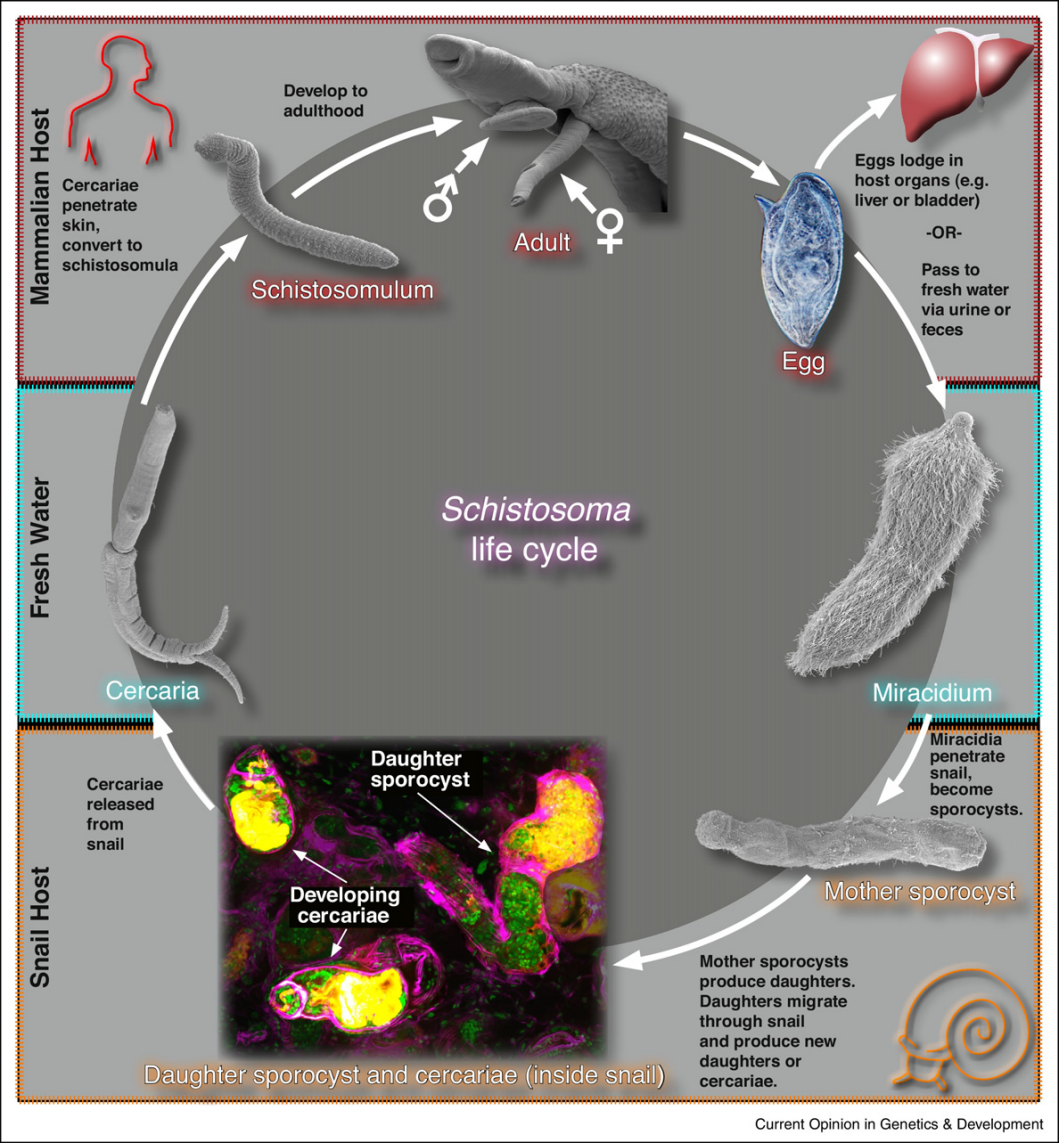
The schistosome life-cycle. See text for details.

Adapted from [52].

Like all trematodes, the schistosome life cycle includes both intermediate (snail) and definitive (mammalian) hosts [19] (Figure 1). The life cycle begins when the eggs shed *via* urine or feces from an infected human reach fresh water. These eggs hatch to release free-living ciliated larvae, known as a miracidia, that proceed to locate and invade a snail intermediate host. Once the miracidium enters the snail, it undergoes a dramatic developmental conversion, becoming another larval stage known as the mother sporocyst. Each mother sporocyst gives rise to hundreds of larvae, termed daughter sporocysts. These daughter sporocysts eventually leave the mother sporocyst and migrate to distal regions of the snail. Here the daughter sporocysts make the developmental decision to produce either new generations of daughter sporocysts or to produce another free-living stage called cercariae. The cercariae will eventually burst out of the snail into the water, where it finds the parasite's definitive host and burrows into its skin. Once in their definitive host, these parasites enter the circulation and begin to develop as either male or female parasites. The male and female worms find each other within the host's circulation, physically attach to one another, and then begin laying eggs. When these eggs traverse the intestine or bladder and are released from the host, the life cycle is completed.

Though this complex life cycle makes study of schistosomes difficult, numerous techniques have been developed in order to facilitate the study of the parasite. Eggs can be cultured *in vitro* and induced to hatch into miracidia *in vitro*, allowing study of this developmental transition. These miracidia can then be either transformed into sporocysts *in vitro* or used to experimentally infect snails, facilitating the study of sporocyst development and maintenance. It is also possible to induce shedding of infective cercariae by simply exposing infected snails to light. Shed cercariae can be mechanically disrupted in order to transform into schistosomulae *in vitro*. Alternatively, mice or other suitable hosts can be experimentally infected with the shed cercariae, and all stages within the definitive hosts can be studied.

If one considers the bizarre nature of this life cycle, it is astounding that these parasites are so successful. In the end, though, their success hinges on two striking developmental attributes: (1) the clonal expansion of sporocysts and (2) the adult parasite's prodigious reproduction that is sustained over the course of several decades. Below we briefly discuss what is known about each of these developmental feats.

## Unique stem cells amplify the schistosome's probability of infection

**Figure 2:**
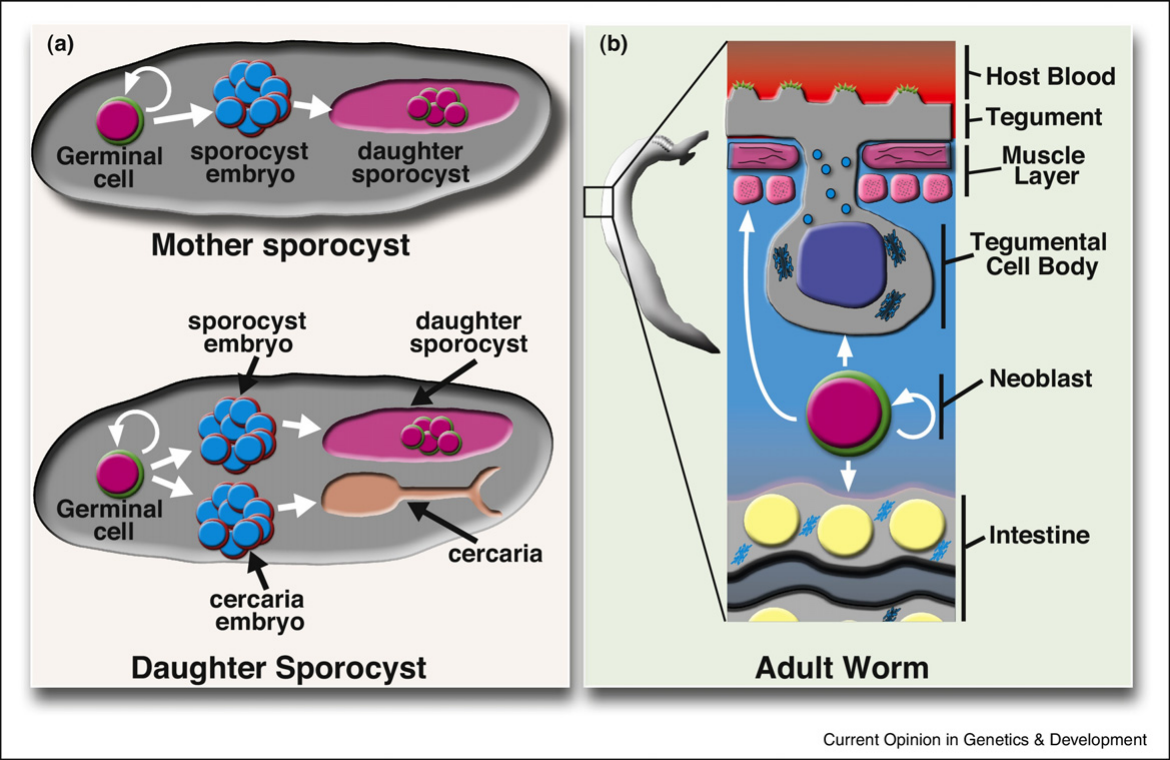
Roles for stem cells in schistosome asexual amplification and adult tissue homeostasis. **(a)** The germinal cells in the mother sporocyst are capable of giving rise to the daughter sporocysts, and germinal cells in the daughter sporocyst are capable of giving rise to more daughter sporocysts as well as infective cercariae. **(b)** The adult neoblasts are capable of self-renewing and giving rise to endodermal (intestinal), mesodermal (muscle), and ectodermal (tegumental) lineages.

Once a miracidium invades a snail and begins producing daughter sporocysts, it has a virtually never-ending capacity to generate infective cercariae. Indeed, it appears that the major factor limiting cercariae production in nature is the life of the snail, since clonal populations of sporocysts can be serially transplanted between snails for many generations, long after the original donor snail would have died [26]. The classic literature credits asexual amplification to a population of cells called germinal cells [27, 28]. Following the conversion of the miracidia to sporocysts, these germinal cells begin a phase of rapid proliferation before undergoing embryogenesis in the absence of fertilization to generate hundreds of daughter sporocysts [28, 29, 30••]. In these daughter sporocysts a similar phase of germinal cell proliferation and embryogenesis ensues, although this time the germinal cells are capable of producing embryos for new generations of either daughter sporocyts or cercariae [28, 31, 32] (Figure 2a).

So what are these germinal cells? Although much larger, germinal cells morphologically resemble the neoblasts of free-living flatworms: they have a high nuclear-to-cytoplasmic ratio, an open chromatin structure, and a large nucleolus [33]. Recent studies also indicate that these cells express factors characteristic of planarian neoblasts, including Argonaute-family proteins and Vasa-like proteins [30^••^]. Interestingly, it was shown that the germinal cells of mother sporocysts exist as two molecularly distinct cell populations which proliferate at different rates. Some germinal cells express a homologue of the RNA-binding protein Nanos while others do not. EdU pulse experiments demonstrated that *nanos*^−^ germinal cells proliferate much more rapidly than those that are *nanos*^+^. The two populations of germinal cells also possess different requirements for canonical stem-cell maintenance factors. Depletion of a vasa-like gene results in a complete loss of both *nanos*^+^ and *nanos*^−^ germinal cells whereas loss of *ago2*, an argonaute homolog, only depletes the rapidly proliferating *nanos*^−^ germinal cells [30^••^]. While the precise fate of these two populations is not known, it could be that one population serves in a ‘stem cell-like’ role whereas the other may represent differentiated progeny, committed to producing the next generation of sporocysts. This bizarre asexual ‘polyembryony’ raises fundamental questions: what molecular programs regulate germinal cell self-renewal and differentiation? Do germinal cells only produce embryos or are these cells able to participate in sporocyst tissue homeostasis and/or regeneration? On a molecular level, do these cells share more in common with somatic stem cells, germ cells, or early embryonic cells? What distinguishes between the germinal cells in the mother sporocyst and in the daughter sporocyst? What is the nature of the schistosome ‘germ line,’ and is it specified in sporocysts, in cercariae, or during adult maturation? Indeed, the ability of the germinal cells inside of the mother sporocyst to proliferate clonally and give rise to seemingly totipotent daughter sporocysts is an astounding developmental feat that warrants further investigation. With the emerging tool kit to study sporocyst development (RNAi, *in situ* hybridization) there are tremendous opportunities to address this unique and important biology.

## Neoblast-like adult stem cells likely promote schistosome longevity *in vivo*

To ensure the continuity of the life cycle, the female schistosome has evolved as a veritable egg-laying machine, capable of producing an egg every one-minute to five-minutes [29]. Although sustained egg production is key to the parasite's success, it is paradoxically the central driver of pathology. In order to complete the parasite's life cycle, the eggs must pass from the host's circulation into the host's excretory system (either into the lumen of the bladder or into the lumen of the intestine). Despite this, as many as half of the parasite's eggs are never excreted from the host and continue to reside in the vasculature where they eventually deposit in the host's organs (e.g. liver or bladder), evoking potent inflammatory responses that can lead to hepatic fibrosis, portal hypertension, splenomegaly, and in some cases, even cancer [34, 35]. In fact, parasites incapable of egg production produce no significant pathology in their host.

In conjunction with their robust egg production, schistosomes are also capable of surviving for decades inside their host; the literature is rife with cases of patients harboring reproductively active schistosomes 20–30 years after leaving endemic regions [36, 37, 38]. How these parasites flourish for years in what has been described as the ‘most hostile environment imaginable [39]’ (i.e., the host's circulation) remains an open question. It has recently been suggested that the schistosome's longevity may be due in part to a population of previously uncharacterized somatic stem cells [14^••^]. By labeling adult parasites with thymidine analogs, it was demonstrated that these cells have the capacity for both self-renewal and differentiation. These cells, like the germinal cells in sporocysts, also appear to resemble planarian neoblasts. Like planarian neoblasts, the schistosome's proliferative somatic cells possess classic neoblast morphology, are restricted to the mesenchyme, and are not present in differentiated tissues [14^••^]. Similar to the sporocyst germinal cells, the adult somatic stem cells express factors characteristic of planarian neoblasts such as an argonaute homologue and fibroblast growth-factor receptors [14^••^]. Interestingly, genes encoding ‘germline’-associated post-transcriptional regulators that typify planarian neoblasts (i.e., PIWI, VASA, TUDOR) appear to be absent from schistosomes [14^••^]. Instead schistosome neoblasts expresses a homolog of the germline-associated post-transcriptional regulator *nanos* [14^••^] that does not appear to be associated with planarian neoblasts. Although the function of these schistosome neoblast-expressed factors remains largely unexplored, one fibroblast growth-factor receptor, *fgfra*, is ubiquitously expressed in somatic stem cells (as demonstrated by EdU incorporation and FISH) and is required for their maintenance [14^••^]. Through pulse-chase experiments with the thymidine analog EdU it has been shown that schistosome neoblasts are capable of differentiating into mesoderm-derived muscle cells and endoderm-derived gut cells [14^••^]. In more recent work examining the transcriptional profile of post-mitotic neoblast progeny it appears the neoblast's primary role is contributing new cells to the schistosome's surface coat, a structure called the tegument [40^••^] (Figure 2b). The tegument is an uninterrupted syncytium covering the entire outer surface of the schistosome (and all other Neodermata) [39, 41]. Since it serves as the primary barrier between the parasite and the host's circulation, the tegument is presumed to be a key evolutionary adaptation for immune evasion in schistosomes [39, 41]. As such, the observation that neoblasts are important for the maintenance of the tegument suggests that further studies on this neoblast-to-tegument differentiation process could provide new insights into how these parasites evade the host immune system. Presently, the tools to study these worms are limited to *in vitro* approaches, but technological advances will soon allow perturbation of neoblast function and examination of the consequences on the parasite in the context of a natural infection.

## Regeneration and developmental plasticity in adult schistosomes

The presence of neoblasts in adult schistosomes begs the question: can these parasites regenerate following amputation, similar to planarians? Unfortunately, since *in vitro* culture systems fail to fully replicate the parasite's niche inside their host, this is a very challenging question to answer definitively, and conflicting reports exist. In 1956, Alfred Senft and Thomas H. Weller (the latter of whom won the Nobel Prize in Physiology or Medicine for culturing the polio virus) reported posterior regeneration of four amputated worms over the course of 10–20 days in *in vitro* culture [42]. However, this result conflicts more modern studies where *in vitro* cultured parasites were able to rapidly heal wounds but failed to regenerate following amputation [43]. Studies in our own lab have also failed to observe the regeneration of amputated parasites cultured *in vitro* (J Collins, unpublished communication). While these conflicting observations could be chalked up to differences in *in vitro* culture conditions, from an evolutionary standpoint it is not clear why schistosomes would possess the ability to regenerate following amputation since they would never encounter this type of insult in the host. A more systematic examination of the response of schistosomes to various types of physical wounding may yield interesting results.

**Figure 3:**
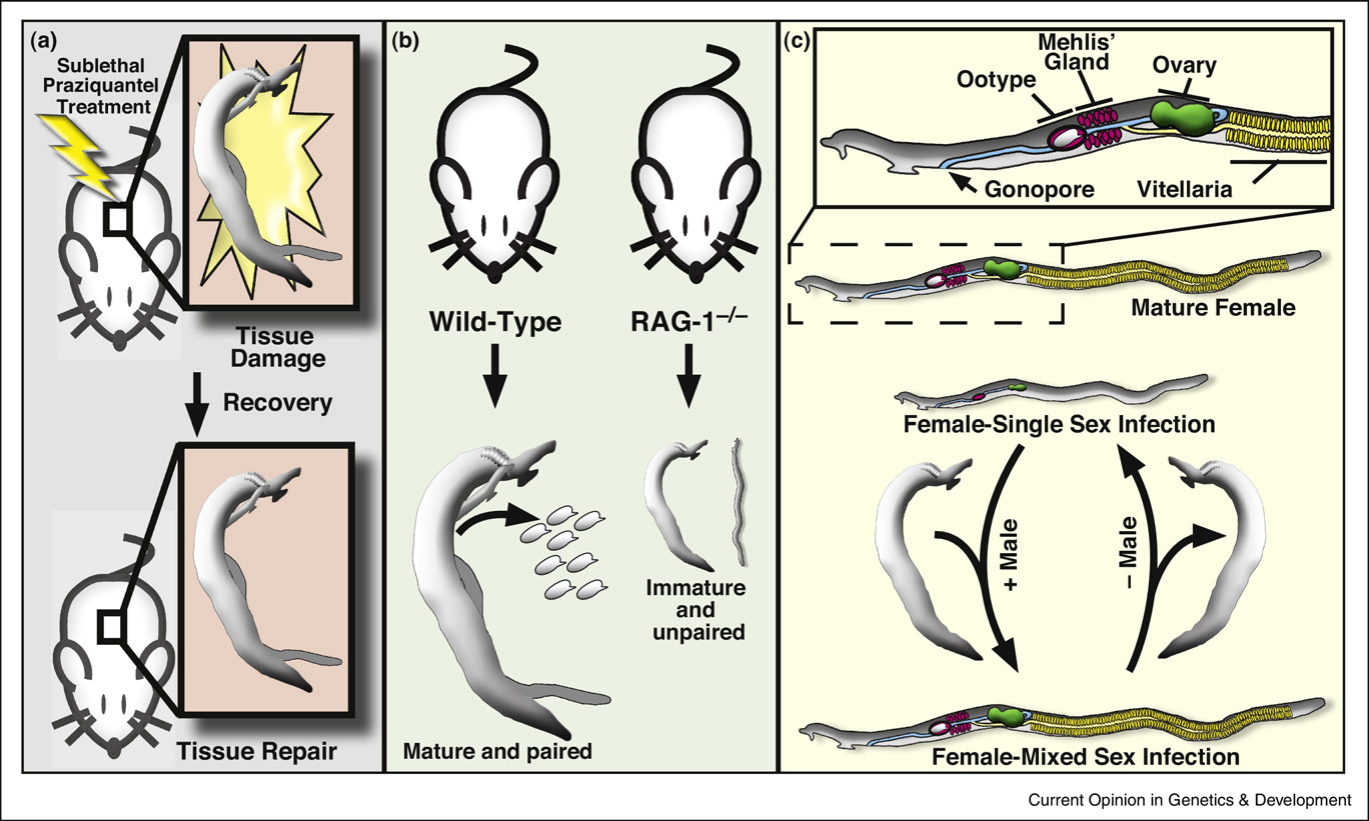
Examples of regeneration and developmental plasticity in adult schistosomes. **(a)** Schistosomes are able to at least partially regenerate their tegument following sub-lethal praziquantel treatment. **(b)** Maturation and pairing of male and female schistosomes requires an intact immune system. **(c)** Single-sex infection and unpairing of worms results in regression of reproductive organs in the female. This regression is reversible, with restoration of reproductive machinery upon pairing with a male worm.

Even though adult schistosomes are unlikely to experience insults mimicking amputation *in vivo*, they are likely to be on the receiving end of a barrage of chemical and cellular insults (e.g., xenobiotic stress or immune attack). In support of this idea, the literature suggests schistosomes can initiate regenerative responses following these types of toxic stimuli. For instance, treatment of schistosome-infected mice with sub-curative doses of praziquantel results in severe damage to the parasite's tegumental surface, tegumental cell bodies, and underlying tissues [44] (Figure 3a). Although structures on the tegumental surface (e.g., spines) are slow to be repaired, tegumental cell bodies and mesenchymal tissues return to normal within one to two weeks [44]. These observations not only point to the regenerative potential of adult schistosomes, but they also implicate the parasite's regenerative response as a potential mechanism by which parasites could evolve resistance to drugs like praziquantel. The role of neoblasts in the regenerative response to praziquantel is not currently known. However, if neoblasts are found to contribute to this regenerative response, one could imagine targeting neoblasts as a means to enhance the effectiveness of drugs like praziquantel in the treatment of schistosomiasis [34].

In addition to mounting regenerative responses to injury, schistosomes have also evolved sophisticated programs which allow them to match their developmental trajectories with their surroundings in the host's circulation. For instance, schistosomes grown in certain immunodeficient mice (e.g., RAG-1^−/−^) are developmentally stunted, incapable of mating, and thus produce few eggs [45^•^] (Figure 3b). This would appear counter intuitive, since one would anticipate that a fully functional immune system would be an impediment to parasite survival within the host. However, this is likely a strategy to ensure reproductive success. Schistosome eggs must pass from the blood through the endothelium and into the lumen of either the intestine or the bladder, and this process appears to depend on a functional host immune response [46, 47]. Thus, by sensing the immune status of their host and adjusting their developmental outputs accordingly, schistosomes can avoid producing eggs when it is unlikely that they would be capable of passing into the environment and completing the lifecycle. Schistosomes also control their development based on the presence or absence of worms of the opposite sex. It was observed nearly a hundred years ago that female parasites from infections containing no male worms are small in stature and their reproductive organs are undeveloped [48^•^] (Figure 3c). Interestingly, this developmental arrest is reversible, since the hypotrophic reproductive organs of female parasites deprived of their male counterpart regenerate if pairing with a male is reestablished [49]. Unfortunately, there are few mechanistic details that explain how either the host immune system or female pairing-status regulates parasite development. We hypothesize that the regulation of stem cell behavior (i.e., proliferation and differentiation) plays a key role in these processes and represents yet another aspect of basic stem cell biology ripe for study in this pathogen.

## Concluding remarks

Stem cells are clearly playing several important roles the biology of these parasites. As alluded to above, an emerging theme in the studies of somatic stem cells from schistosomes is that these cells share multiple fundamental similarities to the neoblasts of free-living flatworms, most notably planarians [14••, 30••]. Since neoblast-like cells play central roles in the complicated life cycles of not only schistosomes but also in a variety of other parasitic flatworms [50, 51, 52•], it is tempting to speculate that neoblast-like stem cells were an important driver in the evolution of parasitism in this group. A single planarian neoblast has the capacity to generate every cell type in the planarian [22]. One could not imagine a better developmental template upon which to evolve the complex life cycles of trematodes like schistosomes or the extreme growth of tapeworms inside their hosts. Indeed, when distilled down to its most essential components one could argue that schistosomiasis (and other diseases caused by flatworms) are fundamentally diseases of stem cells: the germinal cells ensure the success of the lifecycle by amplifying infectivity, the neoblasts promote parasite longevity in the host resulting in chronic illness, and the germ line stem cells generate eggs (and therefore the pathology of the disease). Thus, by asking basic questions about stem cell biology in schistosomes, we can better understand this important disease while simultaneously expanding our knowledge of general stem cell behavior and regulation. We hope that this line of thinking will attract new interest in studying these devastating, albeit fascinating, parasites.

## References and recommended reading

Papers of particular interest, published within the period of review, have been highlighted as:

• of special interest
•• of outstanding interest

## References

[1] Chitsulo L., Engels D., Montresor A., Savioli L. (2000). The global status of schistosomiasis and its control. Acta Trop.

[2] van der Werf M.J., de Vlas S.J., Brooker S., Looman C.W., Nagelkerke N.J., Habbema J.D., Engels D. (2003). Quantification of clinical morbidity associated with schistosome infection in sub-Saharan Africa. Acta Trop.

[3] King C.H. (2010). Parasites and poverty: the case of schistosomiasis. Acta Trop.

[4] Hotez P.J., Fenwick A. (2009). Schistosomiasis in Africa: an emerging tragedy in our new global health decade. PLoS Negl Trop Dis.

[5] Assare R.K., Tian-Bi Y.N., Yao P.K., N’Guessan N.A., Ouattara M., Yapi A., Coulibaly J.T., Meite A., Hurlimann E., Knopp S. (2016). Sustaining control of *Schistosomiasis mansoni* in western Cote d’Ivoire: results from a SCORE study, one year after initial praziquantel administration. PLoS Negl Trop Dis.

[6] Berriman M., Haas B.J., LoVerde P.T., Wilson R.A., Dillon G.P., Cerqueira G.C., Mashiyama S.T., Al-Lazikani B., Andrade L.F., Ashton P.D. (2009). The genome of the blood fluke *Schistosoma mansoni*. Nature.

[7] Liu F., Zhou Y., Wang Z.Q., Lu G., Zheng H., Brindley P.J., McManus D.P., Blair D., Zhang Q.H., Zhong Y. (2009). The *Schistosoma japonicum* genome reveals features of host–parasite interplay. Nature.

[8] Young N.D., Jex A.R., Li B., Liu S., Yang L., Xiong Z., Li Y., Cantacessi C., Hall R.S., Xu X. (2012). Whole-genome sequence of *Schistosoma haematobium*. Nat Genet.

[9] Valentim C.L., Cioli D., Chevalier F.D., Cao X., Taylor A.B., Holloway S.P., Pica-Mattoccia L., Guidi A., Basso A., Tsai I.J. (2013). Genetic and molecular basis of drug resistance and species-specific drug action in schistosome parasites. Science.

[10] Boyle J.P., Wu X.J., Shoemaker C.B., Yoshino T.P. (2003). Using RNA interference to manipulate endogenous gene expression in *Schistosoma mansoni* sporocysts. Mol Biochem Parasitol.

[11] Skelly P.J., Da’dara A., Harn D.A. (2003). Suppression of *cathepsin B* expression in *Schistosoma mansoni* by RNA interference. Int J Parasitol.

[12] Stefanic S., Dvorak J., Horn M., Braschi S., Sojka D., Ruelas D.S., Suzuki B., Lim K.C., Hopkins S.D., McKerrow J.H. (2010). RNA interference in *Schistosoma mansoni* schistosomula: selectivity, sensitivity and operation for larger-scale screening. PLoS Negl Trop Dis.

[13] Cogswell A.A., Collins J.J., Newmark P.A., Williams D.L. (2011). Whole mount in situ hybridization methodology for *Schistosoma mansoni*. Mol Biochem Parasitol.

[14••] Collins J.J., Wang B., Lambrus B.G., Tharp M.E., Iyer H., Newmark P.A. (2013). Adult somatic stem cells in the human parasite *Schistosoma mansoni*. Nature.

[15] Collins J.J., King R.S., Cogswell A., Williams D.L., Newmark P.A. (2011). An atlas for *Schistosoma mansoni* organs and life-cycle stages using cell type-specific markers and confocal microscopy. PLoS Negl Trop Dis.

[16] Liang S., Varrecchia M., Ishida K., Jolly E.R. (2014). Evaluation of schistosome promoter expression for transgenesis and genetic analysis. PLOS ONE.

[17] Rinaldi G., Eckert S.E., Tsai I.J., Suttiprapa S., Kines K.J., Tort J.F., Mann V.H., Turner D.J., Berriman M., Brindley P.J. (2012). Germline transgenesis and insertional mutagenesis in *Schistosoma mansoni* mediated by murine leukemia virus. PLoS Pathog.

[18] Lewis F.A., Liang Y.S., Raghavan N., Knight M. (2008). The NIH-NIAID schistosomiasis resource center. PLoS Negl Trop Dis.

[19] Hyman L. (1951).

[20] Newmark P.A., Sánchez Alvarado A. (2002). Not your father's planarian: a classic model enters the era of functional genomics. Nat Rev Genet.

[21] Reddien P.W., Sanchez Alvarado A. (2004). Fundamentals of planarian regeneration. Annu Rev Cell Dev Biol.

[22] Wagner D.E., Wang I.E., Reddien P.W. (2011). Clonogenic neoblasts are pluripotent adult stem cells that underlie planarian regeneration. Science.

[23•] Laumer C.E., Hejnol A., Giribet G. (2015). Nuclear genomic signals of the ‘microturbellarian’ roots of platyhelminth evolutionary innovation. Elife.

[24] Littlewood D.T.J., Bray R.A., Warren A. (2001). Interrelationships of the Platyhelminthes.

[25] Cable J., Harris P.D. (2002). Gyrodactylid developmental biology: historical review, current status and future trends. Int J Parasitol.

[26] Jourdane J., Theron A. (1980). *Schistosoma mansoni*: cloning by microsurgical transplantation of sporocysts. Exp Parasitol.

[27] Cort W.W., Ameel D.J., Van Der Woude A. (1954). Germinal development in the sporocysts and rediae of the digenetic trematodes. Exp Parasitol.

[28] Olivier L., Mao C.P. (1949). The early larval stages of *Schistosoma mansoni* Sambon, 1907 in the snail host, *Australorbis glabratus* (Say, 1818). J Parasitol.

[29] Basch P.F. (1991). Schistosomes: Development, Reproduction, and Host Relations.

[30••] Wang B., Collins J.J., Newmark P.A. (2013). Functional genomic characterization of neoblast-like stem cells in larval *Schistosoma mansoni*. Elife.

[31] Jourdane J., Theron A., Combes C. (1980). Demonstration of several sporocysts generations as a normal pattern of reproduction of *Schistosoma mansoni*. Acta Trop.

[32] Theron A., Jourdane J. (1979). Sequence de Reconversion des Sporocystes de *Schistosoma mansoni* Producteurs de Cercaires, en vue de la Production de N ouvelles Generations de Sporocystes. Z Parasitenkd.

[33] Pan S.C. (1980). The fine structure of the miracidium of *Schistosoma mansoni*. J Invertebr Pathol.

[34] Pearce E.J. (2013). Parasitology. Rejuvenation through stem cells. Nature.

[35] Ross A.G., Bartley P.B., Sleigh A.C., Olds G.R., Li Y., Williams G.M., McManus D.P. (2002). Schistosomiasis. N Engl J Med.

[36] Harris A.R., Russell R.J., Charters A.D. (1984). A review of schistosomiasis in immigrants in Western Australia, demonstrating the unusual longevity of *Schistosoma mansoni*. Trans R Soc Trop Med Hyg.

[37] Hornstein L., Lederer G., Schechter J., Greenberg Z., Boem R., Bilguray B., Giladi L., Hamburger J. (1990). Persistent *Schistosoma mansoni* infection in Yemeni immigrants to Israel. Isr J Med Sci.

[38] Payet B., Chaumentin G., Boyer M., Amaranto P., Lemonon-Meric C., Lucht F. (2006). Prolonged latent schistosomiasis diagnosed 38 years after infestation in a HIV patient. Scand J Infect Dis.

[39] McLaren D.J., Brown K.N. (1980).

[40••] Collins J.J., Wendt G.R., Iyer H., Newmark P.A. (2016). Stem cell progeny contribute to the schistosome host–parasite interface. eLife.

[41] Skelly P.J., Wilson R.A. (2006). Making sense of the schistosome surface. Adv Parasitol.

[42] Senft A.W., Weller T.H. (1956). Growth and regeneration of *Schistosoma mansoni* in vitro. Proc Soc Exp Biol Med.

[43] Popiel I., Irving D.L., Basch P.F. (1985). Wound healing in the trematode *Schistosoma*. Tissue Cell.

[44] Shaw M.K., Erasmus D.A. (1987). *Schistosoma mansoni*: structural damage and tegumental repair after in vivo treatment with praziquantel. Parasitology.

[45•] Davies S.J., Grogan J.L., Blank R.B., Lim K.C., Locksley R.M., McKerrow J.H. (2001). Modulation of blood fluke development in the liver by hepatic CD4+ lymphocytes. Science.

[46] Cheever A.W., Eltoum I.A., Andrade Z.A., Cox T.M. (1993). Biology and pathology of *Schistosoma mansoni* and *Schistosoma japonicum* infections in several strains of nude mice. Am J Trop Med Hyg.

[47] Doenhoff M., Musallam R., Bain J., McGregor A. (1978). Studies on the host–parasite relationship in *Schistosoma mansoni*-infected mice: the immunological dependence of parasite egg excretion. Immunology.

[48•] Severinghaus A.E. (1928). Sex studies on *Schistosoma japonicum*. Quart J Microsc Sci.

[49] Clough E.R. (1981). Morphology and reproductive organs and oogenesis in bisexual and unisexual transplants of mature *Schistosoma mansoni* females. J Parasitol.

[50] Collins J.J., Newmark P.A. (2013). It's no fluke: the planarian as a model for understanding schistosomes. PLoS Pathog.

[51] Brehm K. (2010). *Echinococcus multilocularis* as an experimental model in stem cell research and molecular host–parasite interaction. Parasitology.

[52•] Koziol U., Rauschendorfer T., Zanon Rodriguez L., Krohne G., Brehm K. (2014). The unique stem cell system of the immortal larva of the human parasite *Echinococcus multilocularis*. Evodevo.

